# Viral Causality of Human Cancer and Potential Roles of Human Endogenous Retroviruses in the Multi-Omics Era: An Evolutionary Epidemiology Review

**DOI:** 10.3389/fonc.2021.687631

**Published:** 2021-10-29

**Authors:** Konstantina Kitsou, Maria Iliopoulou, Vana Spoulou, Pagona Lagiou, Gkikas Magiorkinis

**Affiliations:** ^1^ Department of Hygiene, Epidemiology and Medical Statistics, School of Medicine, National and Kapodistrian University of Athens, Athens, Greece; ^2^ Immunobiology and Vaccinology Research Laboratory, First Department of Peadiatrics, “Aghia Sophia” Children’s Hospital, School of Medicine, National and Kapodistrian University of Athens, Athens, Greece

**Keywords:** evolutionary epidemiology, cancer, viral tumorigenesis, viruses, endogenous retroviruses, Viruses in Human Tumorigenesis

## Abstract

Being responsible for almost 12% of cancers worldwide, viruses are among the oldest known and most prevalent oncogenic agents. The quality of the evidence for the *in vivo* tumorigenic potential of microorganisms varies, thus accordingly, viruses were classified in 4 evidence-based categories by the International Agency for Research on Cancer in 2009. Since then, our understanding of the role of viruses in cancer has significantly improved, firstly due to the emergence of high throughput sequencing technologies that allowed the “brute-force” recovery of unknown viral genomes. At the same time, multi-omics approaches unravelled novel virus-host interactions in stem-cell biology. We now know that viral elements, either exogenous or endogenous, have multiple sometimes conflicting roles in human pathophysiology and the development of cancer. Here we integrate emerging evidence on viral causality in human cancer from basic mechanisms to clinical studies. We analyze viral tumorigenesis under the scope of deep-in-time human-virus evolutionary relationships and critically comment on the evidence through the eyes of clinical epidemiology, firstly by reviewing recognized oncoviruses and their mechanisms of inducing tumorigenesis, and then by examining the potential role of integrated viruses in our genome in the process of carcinogenesis.

## Introduction

The pathogenic effect of viruses has led to the misconception that viruses could only be harmful, with scientific research also being oriented in this direction for many years. Viruses can be perceived as evolutionary champions that seem to play a paramount role in human health and disease as underlined by the increasingly developing study field of the mammalian virome (1). Viruses are linked to extensive phenomena of micro-and macro-evolution, developing a wide range of actions characterized by commensalism, competition, or parasitism and thus, causing respectively the states of symbiosis or dysbiosis with their host. Oncoviruses, a non-taxonomic group of viruses with research history resembling roller-coaster, have either been considered unrelated or even the main cause of cancer. Although we know that viruses are neither necessary nor sufficient to cause cancer, research has led to linking viruses with the development of cancer and the foundation of cancer biology. To date, the worldwide burden of cancers with viral etiology accounts for almost 12% of cancer cases – exceeding 20% in the developing world (2, 3). According to the International Agency for Research on Cancer (IARC), seven viruses have been characterized as Group 1 carcinogens for humans but their part in cancer pathogenesis seems to vary among different viruses and different disease forms (4, 5). The seven carcinogenic viruses along with their route of transmission and their associated malignancies are summarized in **Table 1**. Carcinogenesis is a multi-casual and multi-stage process and viruses, whether active or in latency, seem to act directly, indirectly, and synergistically with multiple co-factors towards the induction of cancer’s hallmarks. While tumorigenesis might be seen as an underachieved adaptation, the preservation of oncoproteins in tumor-viruses, as demonstrated by phylogenetic analyses, suggests the evolutionary advantages of viruses as life forms and the co-evolutionary dynamics in the virus-host interaction during chronic infection leading to carcinogenesis.

**Table 1:** Summary of the viruses classified as Group 1 carcinogens for humans according to the IARC, route of transmission and associated malignancies.

Virus	Genetic Material	Route of Transmission	Associated Cancer Types
EBV	dsDNA	Oral transmission *via* saliva, transfusion (reported)	Burkitt lymphoma, classic Hodgkin’s lymphoma (especially mixed-cellular subtypes), Lymphomas in immunosuppressed individuals (post-transplant and HIV-associated lymphoproliferative disorders), Extra-nodal Natural Killer/T-cell lymphoma (nasal type), Nasopharyngeal carcinoma, Gastric cancer (LD), Diffuse large B-cell lymphoma of the elderly (LD), Lymphoepithelioma-like carcinoma (LD)
HBV	partially dsDNA	Percutaneous and permucosal exposure to infected body fluids, sexual contact, blood and blood product transfusion, solid organ transplantation from an infected donor, unsafe needle practices, vertical transmission	Hepatocellular carcinoma, Cholangiocarcinoma (LD), Hodgkin’s lymphoma (LD), non-Hodgkin’s lymphoma (LD), Pancreatic Carcinoma (LD)
HCV	ssRNA(+)	Blood and blood product transfusion, solid organ transplantation from an infected donor, unsafe needle practices, perinatal and sexual transmission (less effectively)	Hepatocellular carcinoma, non-Hodgkin’s lymphoma (especially B-cell), Biliary tract and Gallbladder carcinoma (LD), Myeloid Leukemia (LD), Thyroid carcinoma (LD)
HHV-8	dsDNA	Oral transmission *via* saliva, parenteral transmission (possible), transplantation (reported)	Kaposi’s sarcoma, Primary effusion lymphoma, Multicenter Castleman’s Disease
HPV	dsDNA	Skin-to-skin contact, skin-to-mucosa contact, perinatal transmission (rare)	Cervical Cancer (HPV:16,18, 31,33,35,39,45,51,52,56,58,59), HPV16: cancer of the vulva, vagina, penis and anus, oral cancer, oropharyngeal carcinoma, tonsillar carcinoma, cancer of the larynx
MCPyV	dsDNA	Skin contact (Not clarified)	Merkel Cell Carcinoma, chronic lymphocytic leukemia (reported)
HTLV-1	ssRNA(+)	Sexual transmission, vertical transmission (mostly through breastfeeding), transfusion of cellular blood products, unsafe needle practices (rare)	Adult T-cell leukemia/lymphoma
HIV-1	ssRNA(+)	Sexual transmission, parenteral transmission (blood and blood product transfusion, unsafe needle practices), vertical transmission (placental, child delivery, breastfeeding)	Kaposi’s sarcoma, non-Hodgkin lymphoma, Hodgkin’s lymphoma, Cervical and anogenital carcinoma, Cancer of the conjunctiva, Cancer of the vulva, vagina, and penis (LD), Skin carcinoma (LD), Lung and Hepatocellular carcinoma (LD)

“IARC monographs on the evaluation of carcinogenic risks to humans, volume 100 B, biological agents” (4) is the source of the information in this table.

LD, Limited Data.

Human studies focusing on the unequal distribution of viruses between cases and control subjects constitute the cornerstone for initial research and have been widely reviewed. Disentangling between cause and effect following these epidemiological observations remains a challenge, one that can be supported by the revolution of high throughput molecular technologies. The first step in the era of –omics was made with the development of genomics, whose roots reside in DNA sequencing and already counts three generations, the latest two known as High Throughput Sequencing (HTS) technologies. HTS allowed the design and successful completion of large-scale cancer genomics projects like the Cancer Genome Atlas and the International Cancer Genome Consortium recording for the first time a detailed list of mutations from tumors and cancer cell lines (6). The development of HTS has provided a more detailed perspective on the understanding of the virus-cancer link, concerning viral types and geographic prevalence of viruses and related malignancies.

Transcriptomic data capture a more dynamic picture and improve the current understanding of many fundamental viral-host pathophysiological processes, including expression of repetitive elements, such as Human Endogenous Retroviruses (HERVs), whose potential implication in human cancer will be examined later in this review. The development of metagenomics widely enables and facilitates the detection of novel viruses, by utilizing sequence information from known pathogens to identify related and still undiscovered agents in samples (7). Of note, metagenomic pipelines have been designed for the detection of viral species and quantification of their gene expression in tumors (8). Epigenomics fills the gap between genomic and transcriptomic and enables the identification of histone modifications catalyzed by acetylation, methylation, and phosphorylation and thus regulate transcription (9).

Thus, the rapid development of HTS technologies in the last decade has enabled the elucidation of multiple mechanisms with which viruses, both exogenous and endogenous, can contribute to the induction of genomic instability to their host and subsequently the development of malignancy. Here, we will firstly focus on the importance of -omics mechanistic studies in elucidating lesser-known mechanisms of viral tumorigenesis while addressing chosen examples of their application and we will discuss the evolutionary implications arising from ongoing research in the human-virus multi-omics research arena regarding recognized carcinogenic viruses. Finally, we will focus on the evolutionary relationship between humans and HERVs, evidence of the potential role of HERVs in human cancer development, and a variety of tumorigenic mechanisms they appear to employ.

## Multi-omics in the Study of Mechanisms of Tumorigenesis of Well-Established Oncoviruses

In this first part of our manuscript, we aim to revisit selected examples of mechanisms by which the seven IARC recognized oncoviruses contribute to the development of malignancy in humans.

### Epstein–Barr Virus

**Figure 1:**
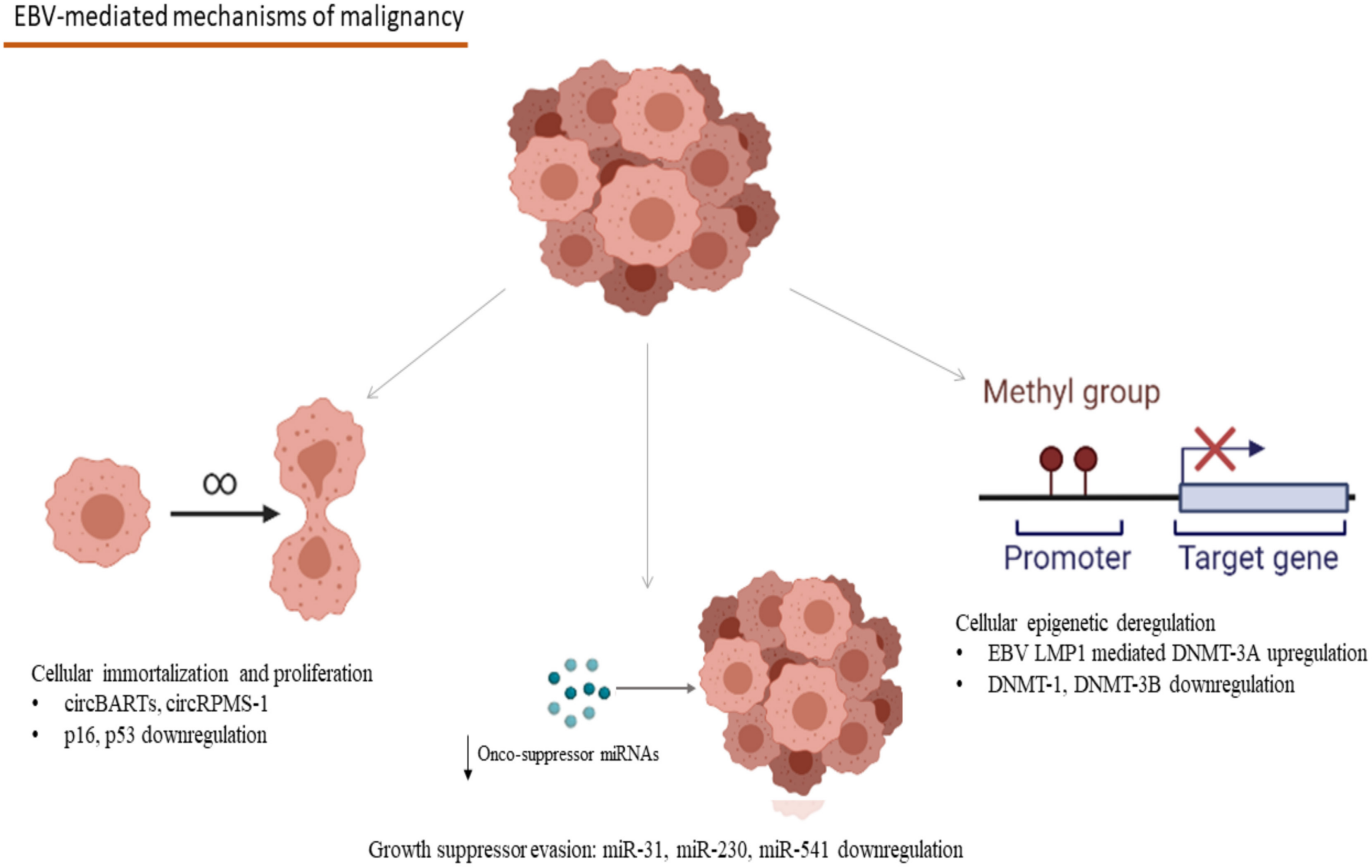
Mechanisms involved in the EBV-associated carcinogenesis: CircBARTs and circRPMS1 are implicated in the cellular immortalization and the induction of uncontrolled proliferation. CircRPMS1 is negatively correlated to the expression of innate oncosuppressor miRNAs (miR-31, miR-230 and miR-541), as observed in NPC. Gene expression modification takes place in B-cell malignancies, as a result of the methylation pattern-modifications caused by the EBV infection through the EBV LMP1 mediated upregulation of DNMT-3A and the downregulation of DNMT-1 and DNMT-3B (EBV, Epstein–Barr Virus; circBARTs, circular BamHI A rightward transcripts; miRNAs, microRNAs; NPC, Nasopharyngeal Carcinoma; EBV LMP1, EBV latent membrane protein 1; DNMT, DNA methyltranserase). Created in BioRender.com.

Concerning the evolutionary aspects of Group 1 tumorigenic viruses, Epstein–Barr Virus (EBV) provides us with an example of human-virus evolutionary conflict. Several EBV microRNAs (miRNAs) have been conserved across ≥13 million years in the Lymphocryptovirus genus, a member of the Herpesviridae family (10), possibly due to a “stealth” advantage they offer over the host’s immune reaction (11). Furthermore, circular RNAs (circRNAs), with not yet clarified functions seem to be preserved through functional homologs among lymphocryptoviruses and appear to play an important role in viral biology, the persistence of infection, and latency stage as well as virus-associated malignancy development (12). Interestingly, EBV circular BamHI A rightward transcripts (circBARTs) were linked to latency in EBV-related tumors including post-transplant lymphoproliferative disease (13) (**Figure 1**).

HTS offered a detailed understanding of the variability of different EBV strains and their geographic distribution (14) explaining the subsequent differences in the geographical distribution of endemic EBV-associated malignancies (15). HTS has enabled the recognition of numerous EBV strains and allowed a detailed understanding of the differences between type 1 and type 2 strains, the most widely used classification for EBV strains (16). Epstein-Barr Nuclear Antigen-2 (EBNA-2) is the main source of sequence variation between type 1 and type 2 strains of EBV, but HTS approaches recently recognized SNPs located in the EBNA-3 viral gene as well as in its nearby gp350 and gp42 viral genes (16). The recognition of further disctinctive characteristics between type 1 and type 2 strains could be proven useful in the disambiguation of the exact mechanisms by which type 2 strains lead to decreased B-cell growth and transformation, reducing its malignant dynamics compared to type 1 strains.

Multi-omics studies have unraveled EBV’s central role in the pathogenesis of Natural Killer/T-Cell Lymphomas (NK/T lymphomas) and Nasopharyngeal Carcinoma (NPC) (17). More specifically, EBNA-1 derived from NPC tumors demonstrated reduced function in the preservation of latency by failing to maintain episomal integrity and thus to suppress the EBV lytic cycle at an epigenetic level (17). Methylation modification seems to take place after the EBV infection of gastric epithelia too, leading to anomalies in enhancer and promoter activity of multiple host genes, which have been observed both *in vitro* and *in vivo*, in EBV-positive gastric malignancies (18). The modifications described in this study were linked to hallmarks of carcinogenesis, including angiogenesis, inflammation, cell migration dynamics, uncontrolled proliferation, and evasion of apoptosis (18). In the case of EBV-related hematologic malignancies, after B-cell infection, EBV remains latent in the germinal centers of secondary lymphoid tissues, and mostly in memory B-cells, where EBV infection appears to affect cellular methylation patterns and has been linked to distinct gene expression profiles (19). EBV infection causes deregulation in the expression of DNA methyltransferases with the upregulation of DNMT3A and downregulation of DNMT3B and DNMT1 (19). More specifically, the upregulation of DNMT3A is mediated through the EBV latent membrane protein 1 (EBV LMP1) oncogene, one that plays a crucial role in the development of Hodgkin’s Lymphoma and the methylation modifications caused in the host’s genome demonstrated a specific pattern, rather than a random distribution, creating specific epigenetic signatures (19) (**Figure 1**). Furthermore, a crucial expressional modification in the process of EBV-related carcinogenesis is the interference of EBV antigens with the regulation of cornerstone proteins in the human cell antitumor defense, like p16 and p53 (20–22). The relation between EBV antigens and these proteins has been extensively studied in B-cell lymphomas like Hodgkin lymphoma, where EBV-mediated downregulation of p16 and p53 has been linked to lymphoblastic proliferation (20–22) (**Figure 1**). The role of epigenetic involvement in the EBV-related carcinogenesis makes epigenetic inhibitors a promising candidate in treating EBV-associated malignancies both *in vitro* and *in vivo* in an animal study with a model of NPC in mice (23).

Despite the unquestionable value of proteomic approaches, they do not appear to be a proper or sufficient approach for the elucidation of the EBV-related carcinogenesis due to the great variety of the pathological events involved in tumorigenesis related to this virus (24). Comparative proteomics is a layout commonly used in this case in order either to compare proteome in different cycles and stages of infection (25) or even to compare proteomes of cell lines infected with other herpesviruses (26). Clarification of virus-related immunogenomic features of various tumors could facilitate the development of immunotherapy by targeting virus or virus-induced proteins that could sustain a feasible therapeutic target.

### Kaposi Sarcoma Herpesvirus/Human Herpesvirus-8

Kaposi Sarcoma Herpesvirus/Human Herpesvirus-8 (KSHV/HHV-8) appears to be a slowly evolving virus with a substitution rate estimated at 6.42 ×10^−4^ substitution/site/year and a low mutation rate comparable to othe double stranded DNA viruses, lower than RNA viruses (27–29). This fact however could be altered as a result of the increase in Human Immunodeficiency Virus (HIV) prevalence that led to a subsequent Kaposi Sarcoma (KS) epidemic (30). Despite the emergence of KSHV/HHV-8 malignancies in the setting of the HIV epidemic, non-HIV-related KS, despite rare (31), underscores the role of the hosts’ innate characteristics along with their immune condition in the KS development.

HTS technologies have aided the elucidation of the multiple mechanisms by which KSHV/HHV-8 leads to either KS or B-lymphoproliferative malignancies, i.e. Primary Effusion Lymphoma (PEL) and the plasmablastic variant of multicentric Castleman’s disease (MCD). Alike EBV, viral cirRNAs are expressed in PEL cell lines and appear to play an important role in the process of carcinogenesis induced by DNA viruses (13). The presence of HHV-8/KSHV encoded circRNA from the vIRF4 viral locus (circvIRF4) as well as RNase-R resistant Polyadenylated Nuclear (circPAN) has been recognized in HHV-8/KSHV infected PEL cells (13). Interestingly, RNase R-resistant circvIRF4 has been recognized in latent PEL cells (13). Conversely, the expression of circPANs is upregulated during HHV-8/KSHV lytic replication (32). The exact mechanisms by which circRNAs encoded by HHV-8/KSHV result in the development of the hallmarks of cancer is still little understood. Bearing in mind the disruption mediated by vIRF4 in the p53 and Notch pathways (33) and the effect of long non-coding PAN on angiogenenesis (34), it is reasonable to assume a potential regulatory function of this noncoding RNA on those pathways.

Furthermore, virally encoded proteins, like Latency Associated Nuclear Antigens, viral Interleukin-6, viral chemokines, viral cyclin, and Kaposins have been associated with the balance between lytic and latent phases of the infection and most importantly with the induction of malignancy hallmarks in the KSHV/HHV-8-associated malignancies (35, 36). In RNA-seq analyses accompanied by methylation studies, KSHV/HHV-8 has been shown to promote initially hypermethylation followed by slower hypomethylation in many genomic sites in PEL (37). Three hypomethylated promoters were recognized that led to the subsequent upregulation of Mucin-13 (MUC13), Galectin-1 (LGALS1), and Protocadherin Beta-5 (PCDHB5) genes in PEL cells (37). Increased expression of MUC13 and PCDHB5 have been correlated to epithelial tumors (38–40) and most interestingly, Galectin-1, encoded by LGALS1 participates in the malignant transformation and metastasis of many tumors and was correlated to neoangiogenesis and cancer development in animal models of KS (37). Intriguingly in the same study, the *in vitro* treatment of PEL cells with demethylating agents was shown to slow down cellular growth offering a potential therapeutic target for this KSHV/HHV-8-associated malignancy (37). Furthermore, unfolding the intracellular trafficking of K15, an HHV-8 kaposin mainly expressed during the lytic cycle, could assist pharmaceutical research by elucidating the various integration sites corresponding to the lytic or latent cycle and thus provide the opportunity of specifically targeting integration sites (41, 42).

### Hepatitis-B Virus

Data emerging from sea depths from a family of non-enveloped Hepatitis B Virus (HBV)-related fish viruses indicates that the evolutionary history of hepadnaviruses is older than 400 mya (43). Paleovirological samples verify that HBV infected humans at least 7000 years ago (44). Intriguingly, the exact evolution of HBV over time is still unclear as an HBV sample isolated from a mummy of the 16th century was found to be closely related to modern type D HBV and thus the molecular-clock method for the estimation of HBV origin cannot be utilized, until more ancient paleovirological samples become available and aid to the better comprehension of HBV-human co-evolution (45).

Genomics studies have pointed to the crucial role of the integration profile of viruses in the determination of the cell evolution towards malignancy and the prognosis of certain types of cancer. In the case of HBV-related Hepatocellular Carcinoma (HCC), increased viral DNA integrations and elevated viral load has been linked to the development from chronic hepatitis to HCC (46). Also, HCC tissues demonstrate a significantly higher number of HBV DNA integrations in comparison to tissues derived from the rest of the HBV-infected liver tissues, a difference that could also be detected at a transcriptional level (46). Furthermore, the HBV integration sites are characterized by chromosomal instability, a milestone in the cellular induction of cancer (47). Genomics studies on human Tumor Necrosis Factor α have linked certain polymorphisms to increased HBV-related HCC susceptibility, underlining the importance of the hosts’ intrinsic characteristics on the human-virus interactions (48). Recently, increased expression of the Small Protein of the HBV Surface Antigen has been linked to aberrant transcription factors production, leading to unfavorable prognosis, as it has been linked to increased cell migration and metastatic dynamics, and targeting its expression may offer improvement of survival potentials (49). HBV short mRNAs expressed by HBV-associated HCC were found to produce epitopes that can be recognized by HBV-specific B-cells (50). Autologous T-cells were engineered to express T-cell Receptors specific for these HBV-encoded epitopes and were administered to two patients with metastatic HBV-associated HCC and resulted in the decreased size of their lung metastases (50). These HTS based advancements offer very promising novel therapeutic options for virus-induced malignancies. Methylation mechanisms also sustain possible therapeutic targets for HBV-related liver malignancies. A recent meta-analysis, which examined the methylation modification patterns in HBV-related HCC patients in comparison to HBV-negative HCC revealed hypermethylation in multiple genes, that was correlated to the HBV infection (51). Interestingly, one of those genes (GSTP1) is hypermethylated, not only in HBV-positive HCC tissues but also in the adjacent HBV-infected liver tissues, a fact that underlines the effect of the viral trigger itself in the methylation pattern modifications that characterize HCC (51). Furthermore, these methylation patterns appear to have different geographical distributions which may indicate the effect of differences in the usual HBV genotypes per location (51). Moreover, methylation of HBV’s covalently closed circular DNA is a potential antiviral target, as it was found to markedly reduce viral gene transcription and genome replication, leading to the promotion and maintenance of chronic infection (52).

The implementation of the HBV vaccine in the National Vaccination Schedules of multiple countries shows promising results in terms of HCC prevention and the incidence of HCC has been reduced (53, 54). Impressive reduction in the incidence and mortality of HCC have been reported in Taiwan, and similar effects are expected to occur in the countries were population-wide HBV vaccinations have been established (55). The importance of the HBV vaccination is underscored by the fact that new chronic HBV infections have been associated with vertical transmissions and incomplete vaccination in the affected individuals (56). Additionally, with the increase in the frequency of the initiation at birth of the HBV vaccination, the incidence of chronic HBV infections is expected to decrease in the future and thus HCC cancer incidence (53).

### Hepatitis-C Virus

The hypothesis of the zoonotic origin of Hepatitis-C Virus (HCV) remains debatable. The tendency of viral variants to infect and replicate into hepatic cells to avoid immune responses to the detriment of hepatic tissue is predominant among viruses in the genus of hepacivirus that can establish chronicity (57). A wide range of studies focuses on E1 and E2 envelope glycoproteins which are responsible for its fusion process (58). Although the exact mechanism remains unknown, accumulating evidence suggests some degree of molecular coevolution among those glycoproteins to achieve both the viral fusion to the host’s cell and immune evasion (59).

RNA-sequencing analyses elucidated innate immunity responses, regarding type I interferon expression and inflammatory signatures unriddling immune mechanisms of spontaneous resolution of HCV infection (60). The exact mechanisms by which HCV-related liver cirrhosis in the frames of chronic HCV infection participates in the development of HCC are largely unknown. Recent *in vitro* evidence suggests that signal transducer and activator of transcription 3 (STAT-3) activation and subsequent transcriptional regulation promotes cell survival in HCV-related cirrhosis, providing the potential median link in the process of HCC following cirrhosis in HCV chronic infection (61).

Moreover, HCV exploits DNA methyltransferases to downregulate onco-suppressing genes resulting in cell cycle acceleration and consequently in the production of more viral particles leading to more extensive HCV-related liver damage (62), which could even directly be connected to cell transformation to malignant. On the other hand, HCV-related HCC is characterized by a distinct genome-wide gene enrichment profile, that does not correlate to viral copy burden but rather to genomic hypomethylation (63).

### Human Papillomavirus

Papillomaviruses infect both humans and animals, with recent identification in fish implying an origin of 500 My ago (64). Human Papillomavirus (HPV) escapes immune with intra-epithelial life cycles, lack of viremia during the infection and cell lysis, and low levels of viral protein expression (65). Unique epithelial tropisms of various HPV subtypes are linked to the host’s evolutionary acquisitions, like loss of fur (66). A phylogenetic analysis of Alphapapillomavirus revealed mutations of E6 and E7 facilitating the generation of lower-risk genotypes, a transition favored in the frameworks of viral adaptation and survival, with well-preserved regions being associated with high-risk subtypes, underscoring the role of these well-preserved regions in the processes of tumorigenesis (67).

Regarding the use of HTS in clinical practice and decision making, HTS has elucidated the role of specific viral subtypes in the risk of developing epithelial cancers and markedly contributed to the classification of HPV subtypes as high-risk and low-risk HPVs (68). One of the most important mechanisms by which HPV promotes carcinogenesis is the interference of the E6 and E7 endogenous viral proteins with the epithelial cell apoptosis, immortalization, and consequent genomic stability and inflammatory angiogenesis, through inactivation of p53, retinoblastoma protein, and inflammatory cytokine induction like Interleukin-8 (69). Aside from the viral malignant potential, the hosts’ genetic background is a very important component influencing the malignant dynamics of viruses as differences in skin virome between healthy subjects and patients with Dedicator of cytokinesis-8 (DOCK-8) deficiency, a rare primary immunodeficiency characterized by reduced lymphocyte-mediated immunity in collagen-rich tissues leads to proneness to infections, atopy and HPV-related squamous cell malignancies (70).

HPV DNA methylation can be considered a hallmark of the shift from HPV infection to pre-cancer and thus methylation assays show promising results as potential diagnostic tools for the recognition of women with high-grade premalignant cervical lesions/cervical squamous intraepithelial neoplasia, and these methylation modifications are considered as hallmarks of the transition of HPV-infected epithelium to cervical pre-cancer and subsequently cancer (71). Strong associations were described for alpha-7 genotypes of HPV, an observation that underlines the viral susceptibility to these patterns and the development of malignancy (71). Proteomics approaches have also been utilized to identify differential biomarkers in HPV-infected cancer cells in cervical and head and neck cell lines (72, 73).

Currently, three types of HPV vaccines are available, a bivalent, against HPV16 and 18 - a quadrivalent, against HPV6, 11, 16, and 18 – and most recently a nonavalent, which offers coverage against HPV6, 11, 16, 18, 31, 33, 45, 52 and 58 (74–76). To date, available data on the implementation of vaccination programs against carcinogenic HPV types 16 and 18, show that vaccination programs have led to significant reductions in the incidence of HPV-related cervical lesions, including high-grade squamous cell intraepithelial lesions, squamous cell carcinoma, and adenocarcinoma in situ, and vulvar cancer and pre-cancer (77–80). Furthermore, smaller reductions in the incidence of cervical lesions have been reported in unvaccinated women as well, indicating possibly an effect of herd immunity due to overall reductions in carcinogenic HPV infections in the community (81). In the era of the implementation of HPV vaccinations, when the elimination of cervical cancer as a major public health issue is feasible, the genotype replacement by HPV types not included in the available vaccines is an ongoing subject of research and there is no clear evidence suggesting toward this direction (82, 83), However, according to a transmission model by Man et al, it is still early to make clear inferences about the potential of an HPV genotype replacement, as this will depend on the ecological competition between HPV types in the community, “cross-protection” of vaccine-covered genotypes and natural immunity of the host (84).

### Human Polyomavirus

Two distinct Polyomaviruses, namely JC and BK polyomavirus (JCPyV and BKPyV respectively) were the first to be recognized in humans by two independent groups in the early 1970s (85, 86). Since then, about 100 viral species have been recognized in insects, arthropods, fish, and mammals, 12 of which are found in humans (87, 88). Regarding the evolutionary history of Polyomaviruses, phylogenetic models suggest that it resides back to the last common ancestor of arthropods and vertebrates as it is estimated that at least one polyomavirus was found in this common ancestor (87). The evolutionary pattern of these relatively slowly evolving small DNA viruses resembles the one found in herpesviruses and some retroviruses, where many viral clades occur within the same host species (87).

**Figure 2:**
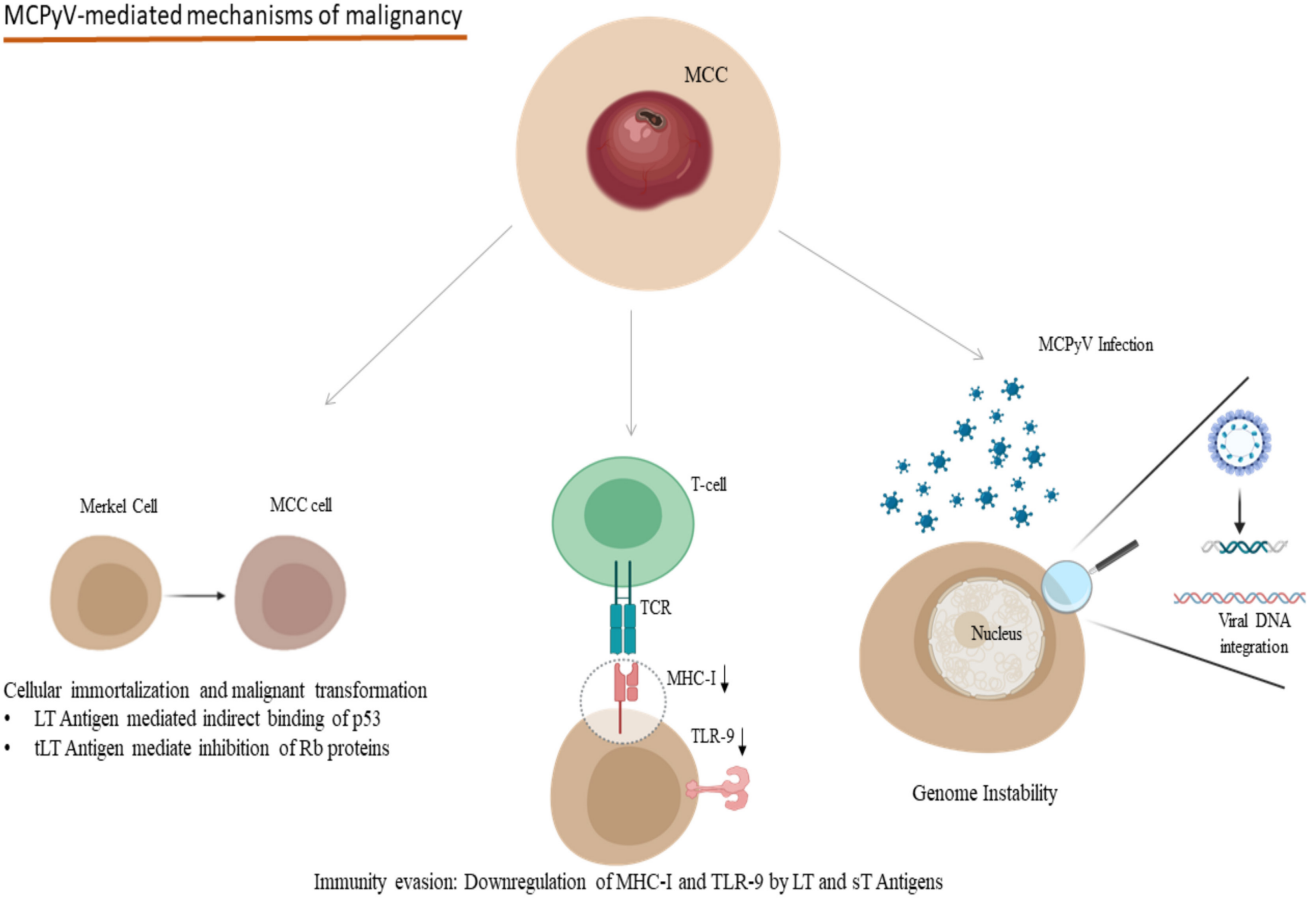
Oncogenic mechanisms in the development of MCPyV-related MCC: Indirect binding of p53 and inhibition of Rb proteins due to LT and tLT antigens of MCPyV lead to cellular immortalization and malignant transformation. LT and sT antigens contribute to the evasion of the immune system mechanisms by downregulating MHC-I and TLR-9. Viral DNA integration to the host genome participates in genome instability. (MCPyV, Merkel Cell Polyomavirus; MCC, Merkel Cell Carcinoma; LT, Large T; tLT, truncated Large T; sT, small T, MHC-I, Major Histocompatibility Complex Class I; TLR-9, Toll-like receptor 9; TCR, T-cell receptor). Created in BioRender.com.

One of the most important polyomaviruses with an oncogenic potential is the recently discovered Merkel-Cell Polyoma Virus (MCPyV), which is found in about 80% of Merkel Cell Carcinomas (MCC) (89) and MCPyV induces its carcinogenic potential mainly through its large T antigen (LT)- full-length LT and truncated LT antigen mutation (tLT) - which is a hallmark of MCC tissue, its small T antigen (sT) and the integration of the viral DNA into the host’s genome (**Figure 2**) (89, 90). The molecular mechanisms of the tumorigenesis mediated by MCPyV infection include the involvement of both full-length LT and tLT antigens in the epithelial cell immortalization and transformation as indicated in rat epithelial cells, the indirect binding of p53 by full-length LT antigen and the suppression of retinoblastoma onco-suppressive proteins (Rb) by tLT antigen (**Figure 2**) (91). Immunocompromised individuals are at increased risk of MCC compared to immunocompetent ones, and transcriptomic studies have revealed the pivotal interference of the LT and sT viral antigens with the host’s defense mechanisms for the virus to escape the host’s innate immunity (92, 93). In particular, LT and sT antigens downregulate the expression of Major Histocompatibility Complex Class I (MHC-I) and Toll-like Receptor-9 (TLR-9), though in a reversible manner (**Figure 2**) (92, 93). This downregulation could be reversed after interferon treatment of the infected cells in the case of MHC-I and after silencing LT and sT antigens in the case of TLR-9 (92, 93). Aside from the infection of dermal cells and Merkel cells, MCPyV DNA and a mutated form encoding for a novel truncated form of LT antigen has been recognized in chronic lymphocytic leukemia cells and this study indicates a tropism of MCPyV toward B-lymphocytes and oncogenic potential in these cells too (94). These findings suggest that a proportion of these leukemias could be attributed to the oncogenic effect of MCPyV (94). Along with the implication of MCPyV in the development of cancer, recent evidence suggests a potential role of other polyomaviruses in the process of carcinogenesis. Examples of these include the recognition of JCPyV DNA in the specimens of primary central nervous system malignancies as well as Human Polyoma Virus-7 (HPyV-7) DNA integrations and the expression of their respective LT antigens in thymomas (95, 96).

### Human Immunodeficiency Virus

Deep evolutionary patterns are seen in HIV, whose epidemic resulted from cross-species transmission of Simian Immunodeficiency Viruses (SIV), which have been infecting primates for at least 32,000 years (97). The Virion infectivity factor (Vif), an accessory protein protecting retroviruses from the immune system, is functionally maintained in HIV-1,2 and SIV (98). The Vif of these viruses have distinct recognition sites, but their functionality remains similar, underlying the importance of the development of mechanisms for the effective evasion to the host’s immune system (98).

The quantification of the viral reservoir and the identification of HIV integration sites in cells of HIV infected individuals could assist a deeper understanding of the increased cancer incidence of HIV-1 patients even in the era of highly active antiretroviral therapy (HAART) with which patients achieve undetectable viral loads while simultaneously contributing to the solution of the HIV persistence problem (99–101). A study conducted on a pair of monozygotic twins indicated that HIV may also cause hypermethylation, a fact which could explain the downregulation in p16 expression that can be attributed to the HIV infection (102). Ocular surface squamous neoplasia, a neoplasm related to HPV and is more common in HIV-infected individuals, demonstrates a lack of p16 expression in the majority of HIV-infected patients (103). However, the presence of p16 expression in ocular surface squamous neoplasia in HIV-infected patients predisposed to a worse prognosis (103). All these data underline the delicate balances in the inter-viral interactions with the host and the intricate malignancy mechanisms in a setting, as such.

An impediment on the comprehension of the interactions between human and HIV-1 proteins and thus immune responses to the virus is the presence of Intrinsically Disordered Proteins (IDPs) (104), proteins with disrupted 3D structures that occur at a higher rate in RNA viruses than in eukaryotes and are associated with immune dysregulation and subsequent immune system escaping of the infected cells enabling long term latency (105).

### Human T-Cell Lymphotropic Virus Type-1

The origin of Human T-cell lymphotropic virus type 1 (HTLV-1) is believed to be the interspecies infection of Simian T-lymphotropic Viruses (STLVs) from monkeys to humans in Africa that occurred around 30.000 years ago (106). Interestingly, despite structural differences, all Tax proteins among HTLV-1, -2, -3, and -4 preserve the CBP/p300 binding region (107). Tax appears to be a key player in the development of malignancy mediated by HTLV-1 as it is implicated in the occurrence of almost all of the hallmarks of cancer, including cell cycle dysregulation, cell immortalization and proliferation, as well as inflammation mediated by cytokines and their receptors, but mainly by trans-activating oncogene cellular promoters (108, 109). Moreover, functionally, Tax-1 has been implicated in adverse effects on the host, such as the induction of genomic instability by inhibiting genomic repair mechanisms and thus leading to Adult T-cell Leukaemia/Lymphoma (ATLL) (107). The wide range of pathogenicity among HTLV viruses examined through comparative structural and functional analysis could specify key features, such as genes and consequently interactions related to oncogenicity (107). HTS has shed light on the effects of HTLV-1 proviral integrations on chromatin loops, causing neighboring host genome loci to approach, and thus the host transcriptional paths can be disrupted, an important mechanism of tumorigenesis (110). At a transcriptional level, HTLV-1 Tax protein affects apoptosis regulation through transcription factor NF-κB dysregulation that leads to inactivation of p53 (111).

Hypermethylation has also been involved in HTLV-1-related malignancies. In this case, hypermethylation happens as a result of mutations in genes that serve as epigenetic modulators, namely in the Ten-Eleven Translocation (TET) methyl-cytosine dioxygenase genes as well as in the Mixed Lineage Leukaemia (MLL) gene family (specifically in families TET2 and MLL3) (112). Regarding the TET2 family, the downregulation of its expression has been linked to the aggressiveness of malignancy (113). Finally, the study of HTLV-1 related tumorigenesis with regards to IDPs has offered information on the proteomic changes that HTLV-1 inflicts and participates in cellular malignant divergence (105).

## Anti-Viral Protective Mechanisms, Characterization of Viral Interactions in the Human Flora and microRNAs in Carcinogenesis

Aside from the contribution of HTS in the elucidation of details of the tumorigenesis mediated by the seven recognized oncogenic viruses, it is important as well to mention the role of the development of HTS in the comprehension of human-viral interaction at the level of anti-viral intrinsic mechanisms, the participation of the virome in the structure and ecology of the human flora and the interference of viruses in the transcription regulation of the host.

### Zinc-Finger Proteins and Retroviral Defense

An important regulator of the transcription of retroviruses (both endogenous and exogenous), in the human genome, are Krüppel-associated box domain-containing zinc-finger proteins (KRAB-KZFPs), that despite their unclearly defined function, appear to be a key player in the regulation of retroviral transcription at an epigenetic level, as evolutionary “young” members of this family appear to have a significant inhibitory function against retroviruses (114). The host’s immune system interacts with viral elements and co-evolves with the virus to defend itself both in the short- and long-term, with Zinc-finger Antiviral Protein (ZAP) being an example of those positively selected mechanisms the discovery of which has been enabled by HTS analyses. Recently, KHNYN, a novel human protein has been recognized as an important co-factor to synergize with ZAPs in the inhibition of retroviral GC-rich RNA replication in human cells for HIV-1 (115).

However, under evolutionary pressures RNA viruses have evolved low GC dinucleotide RNA sequences in their genomes, imitating their hosts, to escape ZAP sensing which identifies elevated GC dinucleotide sequences as “foreign genome” and degrades it (116). Moreover, evidence suggests that Zinc-finger CCCH-type containing 11A (ZC3H11A) protein, which plays a crucial role in the human cell survival during stress by maintaining proper RNA nuclear export, is hijacked by nuclear replicating viruses, as they exploit this mechanism for their own exporting and thus their survival (117).

### Microbiota, Virome Balance and Tumor-Viruses

The importance of the balance during host-viral interactions, even in the commensal or symbiotic state is underscored by the recognition of the virome, along with the microbiota in human skin and mucosa. Human microbiome studies have been focused on bacteria but lately, the virome is identified as a key component for the preservation of host genome integrity. Hannigan et al. showed that bacteriophage communities of Colorectal Cancer (CRC) patients generally affect the gut microbiome and suggested that infectious phages provide nutrition, a friendly environment, and space for opportunistic pathogens to grow (118). The correlation of CRC with a distinct virome signature has also been found by other study groups, that concluded that virome could serve as a diagnostic and prognostic marker for CRC, using a taxonomic approach (119).

Intriguingly, metagenomics approaches have contributed to the recognition of hundreds of different novel HPV genomes, for the first time, in patients with deficiency of DOCK-8, an essential protein in T-cell migration through collagen-rich tissues (70), while a Lactobacillus-rich vaginal microbiota profile has been linked to protection against HPV infections, and thus reduced oncogenic burden (120).

Viruses in the commensal state in the skin have also been linked to beneficial effects in the protection against cancer, through the regulation of the host’s immune system. More specifically, against the previous hypothesis that immunosuppression leads to increased risk of skin cancer through HPV infections, recently a novel mechanism has been proposed for this increased skin cancer incidence, as CD8(+) T cellular immunity against commensal HPVs in the skin microbiota suppresses the development of skin cancer in healthy hosts (121). Thus, lack of integrity of T cellular immunity leads to reduced control of malignant cells, and the researchers demonstrated the importance of intact T cell responses in the defense against chemical and ultraviolet-induced carcinogenesis in mice (121).

### Viral and Human microRNA Regulation in Human Malignancy Development

Deep sequencing approaches have been able to link the expression of specific miRNAs to oncogenicity providing a potential diagnostic tool and therapeutic targets (122), an approach rather promising in the case of viral and host miRNAs interaction that is implicated in virus-related oncogenicity as well. In particular, miRNAs are non-coding RNAs that contribute and take part in the regulation of both host and viral gene expression at a post-transcriptional level. Such miRNAs originating by multiple oncogenic viruses have been correlated to procedures crucial in the development of malignancy, such as inhibition of apoptosis and cell survival, cell proliferation, immune system escape, and cell migration (123), while on the other hand cellular miRNAs appear to interact with oncoviruses (123).

The participation of viral miRNAs and their interactions with those that regulate the host’s defense mechanisms has been extensively studied for EBV, the first virus for which miRNA production has been described. Forty-eight miRNAs are encoded by EBV sequences, 40 of which correspond to the BamHI-A region rightward transcript (BART) and the rest to the BamHI fragment H rightward open reading frame 1 (BHRF1) (124, 125). Interestingly with the use of HTS technology, differential expression profiles have been described for different EBV-related malignancies, namely miR-BART11-3p, miR-BART-17-5p, miR-BART19-3p, and miR-BART19-5p increased transcription in B-cell tumors, and miR-BART8-3p, miR-BART-7-3p, miR-BART22, and miR-BART10-3p epithelial EBV-related tumors (126). B-cell malignant transformation in the development of Burkitt lymphoma is at least partially mediated by miR-BART1 and mir-BART16, while the same miRNAs participate in the maintenance of tumor cells, even in the absence of EBV-infection in those, through targeting Caspase 3 (127). Burkitt lymphoma development is also facilitated through the miR-BART6-3-p-mediated downregulation of PTEN, affecting cell proliferation and immortalization and of WT1 and Interleukin-6, affecting the host’s immune responses (128). NPC tumorigenesis and treatment resistance have been linked to miR-BHRF-1 and miR-BART5-3p, by targeting tumor-suppressor protein p53 (129, 130). EBV modifies the profile of miRNA expression, namely miR-31, an miRNA, participating in the cell’s antitumor defense which is downregulated in EBV-associated NPC (131). TRIM8, a key player in Tumor Necrosis Factor α and NF-κΒ signaling, which is implicated in Burkitt Lymphoma and gastric cancer, is targeted by miR-BART16 (132). Furthermore, the interplay between the expression of viral circRNAs and oncosuppressor host miRNAs has been demonstrated by the negative correlation of the expression of the EBV-encoded circRPMS1 and tumor suppressor miRNAs, namely miR-203, miR-31, and miR-451, with the knockdown of circRPMS1, leading to reduction of NPC cell proliferation, induction of apoptosis and inhibition cell invasion, cornerstones in the pathogenesis of malignancy (133) (**Figure 1**). Generally, conservation of miRNA sequences among the gammaherpesvirus group is rare (134). Despite the evidence that suggest that many gammaherpesvirusese share common miRNAs, conservation of miRNA has been demonstrated only in two pairs of gammaherpesvirusese. EBV and rhesus lymphocryptovirus (rLCV) share conserved miRNAs, while rhesus rhadinovirus (RRV) and Japanese macaque herpesvirus (JMHV) share 9 pre-miRNAs (134). The mechanism underlying the evolution of miRNAs of herpesviruses remains elusive, although at least partially it might be explained through the evolution of host target genes. This could be explained through red queen dynamics which means that host and virus remain in balance through deep time evolution.

Regarding KSHV/HHV-8 tumorigenesis, viral miRNAs and aberrant transcription of host’s miRNAs have been implicated. In a cell line with latent KSHV/HHV-8 infection, KSHV-miR-K12-4-3p and KSHV-miR-K8-3p transcription demonstrated no significant difference from PEL cells, but KSHV-miR-K12-6-3p was upregulated, and KSHV-miR-K12-10a-3p downregulated in PEL cells compared to latent infection, a fact that underlines the dynamic viral miRNA expression between different stages in tumorigenesis (135). KSHV/HHV-8 infection, also, leads to the deregulation of important regulatory miRNAs of the host, including the upregulation of miR-708-5p, which induces reduced expression of Caspase-2 and leukemia inhibitory factor, and increased expression of the p53-inhibitor MDM2, through the downregulation of cellular miR-409-5p (135). Common transcriptomic signatures have been recognized between the KSHV/HHV-8 infection and hypoxia, which led to increased transcription of cellular miR-210, a Hypoxia-Inducible Factor target that induces neoangiogenesis and inhibits apoptosis (136). Surprisingly, KSHV-miR-K6-5p is an “agonist” of the tumor-suppressive miR-15/16 family, leading to cell-cycle inhibition (137). This effect has been postulated to offer an evolutionary advantage to the virus by prolonging its host’s survival. The participation of miRNAs in the development of virally induced-HCC has been recognized for HBV and HCV infection. Deep sequencing approaches have revealed the role of HBV-miR-3 in the establishment of HBV infection persistence and latency of the virus in liver cells (138), while this miRNA is recognized as a PTEN inhibitor, which leads to increased cell proliferation (139). Furthermore, cellular miR-522 and miR-523 are significantly upregulated in HBV-positive HCC tissue compared to HBV-negative HCC and their increased levels were correlated to poor outcomes (140). In HCV-induced HCC, *in vitro* studies revealed that despite the modest changes in the levels of cellular miRNAs, the deregulation of miR-1260a, miR-21^∗^, and miR-27a^∗^, as well as miR-483 and miR-1303 interfered with cellular lipid metabolism, while miR-122 is involved in viral persistence, by interfering with interferon- related pathways (141). Moreover, HCV evades the host’s immune system and established latency, the first step in virally-induced tumorigenesis through the upregulation of cellular miR-21 (142). Thus, type I Interferon production is hampered and the TLR signaling pathway is inhibited, leading to ongoing HCV replication (142).

Wide discrepancies have been described on the miRNA signatures of HPV-related oral and oropharyngeal head and neck cancer. Recent, RNA-seq findings pinpoint the role of HPV infection and cellular miR-9 (143). HPV-E6 protein is one candidate up-regulator for miR-9 leading to cellular proliferation and migration in the setting of oropharyngeal and cervical cancers (143). Small RNA-sequencing analysis (sRNA-seq) offers interesting perspectives in the diagnosis of HPV-induced lesions, as sRNA-seq in HPV‐positive samples with cervical precancer was capable to reveal a distinct miRNA profile of 9 miRNAs, namely let‐7b, miR‐9, miR‐15b, miR‐20a, miR‐31, miR‐93, miR‐183, miR‐184, miR‐222 (144). Our understanding of the HPV encoded miRNAs is currently limited. HPV encoded miRNAs have been recognized and appear to take part in HPV-mediated carcinogenesis (145). For example, HPV16-miR-H1-1 targets genes involved in T-cell activation, cellular adhesion and cell migration, cell growth, survival, and proliferation (145). Computational analyses revealed 19 pre-miRNAs (HPV-pre-miRNAs), varying between HPV types (146). The role of miRNAs in the induction of MCPyV-induced malignancy is controversial, as their low-level expression was considered unlikely to participate in viral carcinogenesis (147). However, recently exosome-shuttle miR-375 was found in Merkel cell carcinoma tissue and can be considered as a mediator of malignancy by downregulating p53 (148).

Many studies have demonstrated that cellular miRNA profile is altered after HIV infection and multiple miRNAs target viral sequences functioning as a host defense mechanism against infection (123). Significant upregulations of DNMT3a and DNMT3b, correlated to decreased transcription of miR29 and miR148-152, have been described in aggressive B-cell lymphomas in HIV-positive individuals, indicating aberrant methylation patterns due to miRNA transcription in response to HIV infection (149). Additionally, the other important carcinogenic retrovirus, HTLV-1, has been correlated to miRNA dysregulation of the host both during its asymptomatic carriage and at the presence of ATLL. For example, significant upregulation of miR-451a and downregulation of miR-142-3p in ATLL were reported compared to healthy control samples, which were correlated to THBS4 upregulation and increased cell migration, and invasion (150). A highly conserved miRNA with tumor-suppressor properties, that is involved in the p53 pathway and is decreased in many tumors, miR-34a (151), was found elevated in Peripheral Blood Mononuclear Cells (PBMCs), early after their infection with HTLV-1 as well as in the PBMCs of patients with ATLL, indicating the consistent miR-34a upregulation during the course of HTLV-1 latency and HTLV-1-related malignant transformation (152). Surprisingly, miR-34a upregulation was correlated to increased survival of the HTLV-1-infected T-cells, as its knock-down led to death of the HTLV-1-infected cells (152). NF-κβ pathway activation appears to be important in the maintenance of the expression of miR-34a, and HTLV-1 Tax was proposed by the researchers as a potential mediator of the role of miR-34a in HTLV-1 tumorigenesis, due to its potent role as an NF-κβ pathway activator (152). However, further research is warranted on the pathways that are associated with the miR-34a role in the development of HTLV-1 malignancy, as the expression of HTLV-1 Tax is significantly reduced and almost disappears in ATLL cells (152). These findings underline the role of other not yet recognized molecular pathways related to the role of this miRNA (152). Recently, an sRNA-seq analysis revealed a distinct profile of miRNA expression in the PBMCs of HTLV−1 asymptomatic carriers compared to controls, a fact that may underline their role in latency and malignant hallmark development (153).

## The Enemy Within: HTS Provides Deep Insight Into Human Endogenous Retroviruses

HERVs are retroviral elements in the human genome, that have integrated into our ancient ancestors’ germ-line cells and were preserved through Mendelian inheritance (154). The co-evolution of Endogenous Retroviruses (ERVs) with their jawed vertebrate hosts has taken place for over 450 My (155). The majority of ERV integrations have become replication- and retrotransposition-defective and remain under strict transcriptomic control (156). These ancient pathogens have lost their ability to replicate and re-infect by the accumulation of multiple mutations, including deleterious insertions and deletions, often causing frameshifts as well as homologous recombination events, leading to the creation of solitary Long Terminal Repeats (LTRs) and the total disappearance of the viral gene coding sequences. due to the evolutionary pressure, they imposed on the physiologic functions of their mammalian hosts. Along with their participation in the retroviral integration, the LTRs flanking endogenous retroviruses function also as regulatory elements, with promoter and enhancer function (157). Given the versatile functions of HERV transcripts and proteins in the hosts’ fitness and diseases, HERVs cannot be considered as “junk DNA” as previously perceived, but appear to have multiple implications in hosts’ evolution, health and disease. Their co-option cannot be attributed with confidence to a specific reason but there is a list of candidates linked to evolutionary advantages for the host that could be responsible for their maintenance in the human genome.

In this part of our manuscript, we will review evidence showing that HERVs are linked to a variety of tumorigenic mechanisms such as chromatin structure modification and imprinting, transcriptional modulation, and post-transcriptional regulatory networks.

### HERVs-Exogenous Viruses’ Interplay Along With the Maternal-Fetal Paradox: Insights to Cancer Immune Escape?

One of the most important characteristics of HERVs is their involvement in placentation with a similar protein to mammals’ retroviral-envelope (Env) protein in an independently evolved lizard with placentotrophic reproduction (158). Env protein apart from being co-opted to serve syncytiotrophoblast formation in placentation is also inversely correlated to the ability of ERVs to spread within host genomes and thus offers protection to the developing organisms (159). What is more, *env* transcripts are found mainly in the fetal part of the placenta, while the maternal part remains relatively poor in retroviral expression (160). This observation underlines the benefits of this retroviral presence in the side of the developing embryo, rather than the maternal side.

In particular, two HERV-derived Env-proteins, Syncytin-1, the Env protein of HERV-W, and Syncytin-2, which is encoded by HERV-FRD, have been implicated in the process of proper placentation and cell-cell fusion (161). Both Syncytin-1 and Syncytin-2 contribute to syncytialization by their expression during placentation, leading to the fusion of the villous trophoblast to the syncytiotrophoblast (162). Syncytin-1 is expressed in the extravillous trophoblast and the villous trophoblast, while Syncytin-2 is expressed solely in the villous trophoblast (161). Syncytin-1 and Syncytin-2 function after their ligation to their cognate receptors Na-dependent neutral amino acid transporter 2 (ASCT2) and Major Facilitator Superfamily Domain Containing 2 (MFSD2) (161, 162). The importance of these HERV-derived proteins in the normal placental formation is pointed out by the observed deregulation of Syncytin-1 expression in the setting of placental syndromes, such as pre-eclampsia, probably offering smaller advantages to those fetuses (163) and of Syncytin-2 in chromosomally abnormal trophoblastic tissues, which leads to insufficient syncytialization and subsequent spontaneous pregnancy loss (164). Simultaneously, the overexpression of methyltransferases, recognized in these tissues, implicates an aberrant epigenetic regulation of HERVs (163).

Intriguingly, an Env protein encoded by HERV-Fb1 (ERVH48-1), Suppressyn (SUPYN) has been recognized *in vivo* placental samples and *in vitro* in cell lines as an inhibitor of cell-cell fusion in mammals and its sequence appears to be highly conserved through simian evolution (165). SUPYN is a Syncytin-1-specific inhibitor, with which it shares its cognate receptor (ASCT2) (165). Furthermore, these two proteins share similar cell-type expression patterns and are both expressed in villous and extravillous trophoblast (165). The ligation of SUPYN to ASCT2 has been proposed to serve beneficial functions for the host. Most importantly, SUPYN is hypothesized as a mediator in syncytialization regulation to avoid aberrant placentation (166). Oxygen levels at the maternal-fetal interface have been implicated in the development of pre-eclampsia and reduced SUPYN expression in a state of hyperoxia is presumed to lead to maternal exposure to placental debris, disruptions in the maternal spiral artery remodeling and could lead to the hallmarks of preeclampsia (166).

Endogenous viral elements interfere with immunity and antiviral defense, and their preservation is similar to that of another set of pregnancy-associated genes, the Killer-cell Immunoglobulin-like Receptor (KIR) family whose expression seems to be pathogen-driven (167). There is an increased interest in the association between HERVs and the “paradox of pregnancy”: pregnancy is compared to semi-allograft, but the fetus manages to avoid maternal immunity (168, 169). The disturbance of this balance could lead to a pro-inflammatory cytokine storm and distressing events resulting in miscarriage (170). This “series of unfortunate events” could be attributed to viral infections, a fact that pinpoints that HERVs are under continuous selective pressure. HERV-K expression is suggested to protect pre-implantation embryos from exogenous viral infections which could lead to their preservation due to natural selection (171).

All these findings underscore the importance of HERV-derived Env protein in the successful cellular fusion during placentation, placental integrity, and fetal-maternal health. Thus, it is reasonable to assume that the tolerance of the immune responses to cancer could be at least partially attributed to the upregulation of HERVs expression that is widely observed in a variety of tumors (172).

### Oncoviruses and HERVs Interactions

Oncoviruses seem to regulate HERV expression through different pathways like host immunosuppression, production of viral transcriptional factors, chromatin dysregulation, and epigenetic modifications (173). Especially for HERV-K HML2 (HK2) a wide range of interactions has been described with oncoviruses.

**Figure 3:**
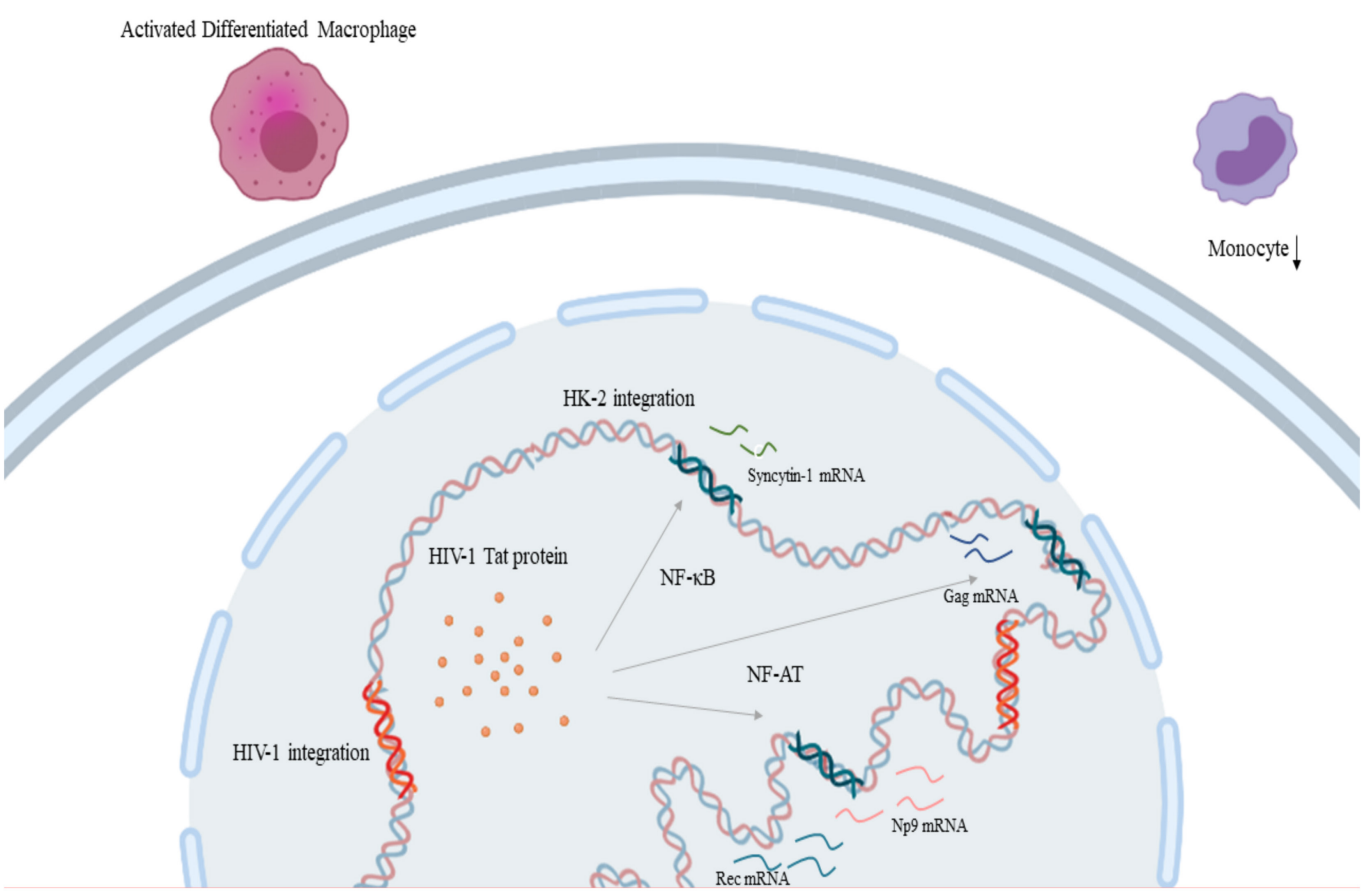
HIV-1 and HERV-K HML-2 interaction in the process of carcinogenesis: HIV-1 Tat protein leads to the upregulation of *rec* and *np9*, and *gag* expression through transcription factors NF-κB and NF-AT respectively. Also, HIV-1 Tat protein leads to the inhibition of monocytes and stimulation in differentiated macrophages through *syncytin-1* expression. (HIV-1, Human Immunodeficiency Virus 1; HERV-K HML-2, Human Endogenous Retrovirus HML-2). Created in BioRender.com.

HIV-1 infection increases expression of HK2 through the Tat protein, as shown *in vitro* when treatment of primary lymphocytes and Jurkat T-cell with recombinant HIV-1 Tat protein led to an upregulation in *rec* and *np9*, and *gag* expression respectively by promoter activation facilitated by NF-κB and NF-AT transcription factors (**Figure 3**) (174, 175) which might contribute to the pathophysiology of cancer development in HIV-1 infection. Moreover, there are indications that HIV Tat protein elaborates a cell-type-specific regulation of *syncytin-1* expression which is translated as inhibition in monocytes and stimulation in differentiated macrophages (**Figure 3**) (176). On the other hand, reduced HIV replication was linked to the expression of HERV-K108’s (a type-2 HERV-K HML-2) Env protein (177). HERV-K18 Env protein demonstrated an ability to be incorporated in HIV but not SIV particles and this interaction between this endogenous and an exogenous retrovirus could be an important mediator in the immune escape of HIV-1 and its carcinogenesis (178). HIV-1 infection, also, at a transcriptional level, leads to the production of a fully glycosylated HK2 Env protein on the surface mononuclear cells of HIV-infected individuals (179). Furthermore, this HK2 Env protein is immunogenic, leading to the production of antibodies against it, the titters of which are particularly elevated in HIV-infected individuals that do not receive treatment (179).

HK2 is transactivated by KSHV/HHV-8 and has been suggested to contribute to the pathophysiology of immunosuppression through Np9, thus contributing to the development of Kaposi’s sarcoma (180). Transactivation of HERV-K18 was noticed in cases associated with EBV (181). EBV has also been shown to upregulate *syncytin-1* expression in astrocytes, B-cells, and monocytes (182). EBV also activates various LTRs through hypomethylation without however this being sufficient to cause carcinogenesis. Subsequently, it was assumed that DNA hypomethylation and transcription factors collaborate towards carcinogenesis (183). Recently, HBV X protein has been associated with the upregulation of *syncytin-1* and the production of both spliced and unspliced transcripts (184). Finally, HTLV-1 Tax is associated with the upregulation of the LTRs and the promoter activity of HERV-H and HERV-W8 sequences, as the use of an HTLV-1 Tax transactivator on Jurkat cells demonstrated similar effect on the T-cell activation with the effect of a potent combination of T-cell stimulating agents: bpV[pic], a cAMP pathway activator, with Forskolin, an inhibitor of Tyrosine phosphatases, and PMA, a protein kinase C activator, respectively (185). Data concerning the remaining oncoviruses are insufficient and remain controversial.

### Evolutionary Pressure on HERVs Through Cancer

The total number of HERVs in the human genome over the past 10 million years has been declining while the acquisition of new and expansion of old families is observed in other species (186). Body size was connected with declining ERVs integrations probably as a potential defense mechanism against tumorigenesis and this is suggested to be relevant to Peto’s paradox, however, this characteristic on its own could not justify the differences mentioned above (187). However, the “golden ratio” of HERVs’ activity for both viral and human survival remains to be established, since some of their “evolutionary virtues” could also be linked to unfortunate events like cancers (172). Of note, viral activity is subject to a lesser degree of transcription control in the elderly in comparison to younger individuals, an important point when considering virus-host interactions under the prism of evolution, with evidence suggesting HERV-K transcription, being found higher in older healthy individuals compared to younger healthy ones (188), an observation that could be correlated to the fact that tumorigenesis is more prevailing in older individuals.

### HERVs, Pluripotency and Cancer Stem Cells

Adult cells can be reprogrammed *via* multiple experimental procedures altering their gene expression patterns (189, 190). Despite the differences regarding the growth factors used to induce pluripotency, these variable procedures share common features and obey the same disciplines when it comes to HERV expression. HERV-H appears to be of great importance, in early events of reprogramming, with the expression of LTR7 in induced Pluripotent Stem Cells (PSC) higher in comparison to Embryonic Stem Cells (ESC) (191). On the other hand, a loss of function study showed that HERV-FRD is a suppressing factor for induced PSC reprogramming (192).

HERVs’ role in the process of carcinogenesis is indicated by their part in cell reprogramming of Cancer Stem Cells (CSC) which are thought to be responsible for tumor initiation, progression, and maintenance, metastatic dissemination, and recurrence (193). HERVs entanglement has been implied mainly due to reports of upregulation after treatment. HERV-WE1, HERV-FRD1, HERV-31, and HERV-V1 have appeared elevated in treatment-naïve colon carcinoma cells after cytostatic interventions (194). Moreover, HERV-H elements were shown to be able to activate Topologically Associating Domains’ (TAD) boundaries in PSCs (195), inducing gene transcription. TAD boundaries are considered able to lead to carcinogenesis when disrupted or rearranged (196). In combination, these indications provide a theoretical framework adept at implying a link among HERVs, pluripotency, and carcinogenesis (197).

Finally, inflammation has variable effects on stem cells. That given, HERVs have been shown to activate immune and inflammatory responses when their RNA is recognized as a pathogen-associated molecular pattern (198). Accordingly, in the case of CSC, HERVs could induce more aggressive types of cancer through inflammation such as in endometrial carcinoma (199) and melanoma (200).

### HERVs and Human Cancer: Link, Therapeutic and Prognostic Perspectives

Unlike cancer development in other mammals, most strikingly in mice, where infectious endogenous retroviruses Mouse Mammary Tumor Virus (MMTV) and Mouse Leukemia Virus (MLV) are the causative agents of the respective malignancies, endogenous retroviral transcription and activity have not been directly and conclusively linked to human malignancy (201). However, HERVs are characterized by multiple traits that make them strong candidates for participating in human tumorigenesis, as it is evident that human carcinogenesis is a rather multifactorial process and, to its greater extend, not yet elucidated. There are many ways that ERVs can be considered an important player in human carcinogenesis. Many HERV LTRs can serve as promoters and enhancers for transcription and affect the regulation of gene expression with this activity more pronounced around gene-loci and transcriptional dysregulation is an important characteristic in cancer development (202–204). One-third of all p53 DNA binding sites are included in HERV sequences (205). To date, evidence points to contradicting roles in cancer in humans. Many ERV families have been linked to increased tumorigenic burden and appear to affect the prognosis to the worst while on the other hand, the immune responses that are mediated through ERV expression like innate immunity upregulation with interferonogenic reactions (206), are found to have cancer-limiting effects in some neoplasias (207). In this chapter, we aim to discuss selected examples of the manifold and multi-faceted effects of HERV expression in the development and prognosis of human cancer.

Many studies have demonstrated a potential implication of ERVs in a wide spectrum of human malignancies including hematologic malignancies and solid tumors. High expression levels of the Env protein of ERV-3-1 were found in all patients with Acute myeloid leukemia (AML) included in one cohort while this effect was more specific for bone marrow and blood cells and was closely associated with a myeloid phenotype of AML in this study (208). Concerning hematologic malignancies, Myelodysplastic syndrome (MDS) is a heterogeneous category of hematological diseases that are recognized predisposition factors for the development of myeloid lineage malignancies, most commonly myeloid leukemia (209). Upregulated expression of ERV-K6, ERV-W1, and ERV-3-1 is associated with the presence of dyserythropoiesis in MDS. The upregulation of these ERVs participates in the induction of this phenotype, by stimulating TLR3 expression resulting in the expression of transcription factors including Interferon Regulatory Factors (IRFs), IRF-3, and IRF-7, as well as NF-κB leading to increased inflammatory burden (209). In contrast, in multiple myeloma, a hematologic malignancy, characterized by methylation pattern dysregulations (210), concomitant inhibition of two distinct methyltransferases, namely EZH2 and G9a, led to ERVs activation by interfering with their methylation-mediated silencing, which in turn stimulated type I interferonogenic reactions (211). This inflammatory response led by ERV transcription, when activated, demonstrated induction of apoptosis and subsequently anti-myeloma effects (211).

Regarding solid tumors, breast cancer is one well-studied paradigm of correlation of HERVs to cancer development. HK2 Env protein co-operates with multiple environmental and host-immune factors and exercises a tumorigenic effect on human breast epithelial cells *in vitro*, by inducing cellular phenotype shift to mesenchymal and increased cell migration and evasion dynamics leading to metastatic potential (212). HK2 serum mRNA and HK2 specific antibodies have been proposed in aiding early diagnosis in breast cancer as their levels in patients with ductal carcinoma *in situ* were significantly higher than in healthy women (213). The levels of these ERV-related biomarkers demonstrated a dose-dependent effect with regards to the progression of tumors and their increase could also predict the occurrence of metastases in breast cancer patients (213). This observed HK2 dysregulation in breast cancer patients is more evident in breast cancer in HIV-infected women, which indicates the retroviral interactions between HK2 and HIV in the upregulation of HK2 in these tumors (214). Lung adenocarcinoma in never-smoker female patients has been linked to polymorphic HK2 integration sites in homozygosity (215). This observation makes the scenario of a potential role of ERVs in human cancer plausible, as this is a rare form of the disease, lacking the usual exposures of this malignancy. Furthermore, metagenomic analyses in the virome of lung cancer tissues and blood from lung adenocarcinoma revealed the presence of multiple HERV families (216).

**Figure 4:**
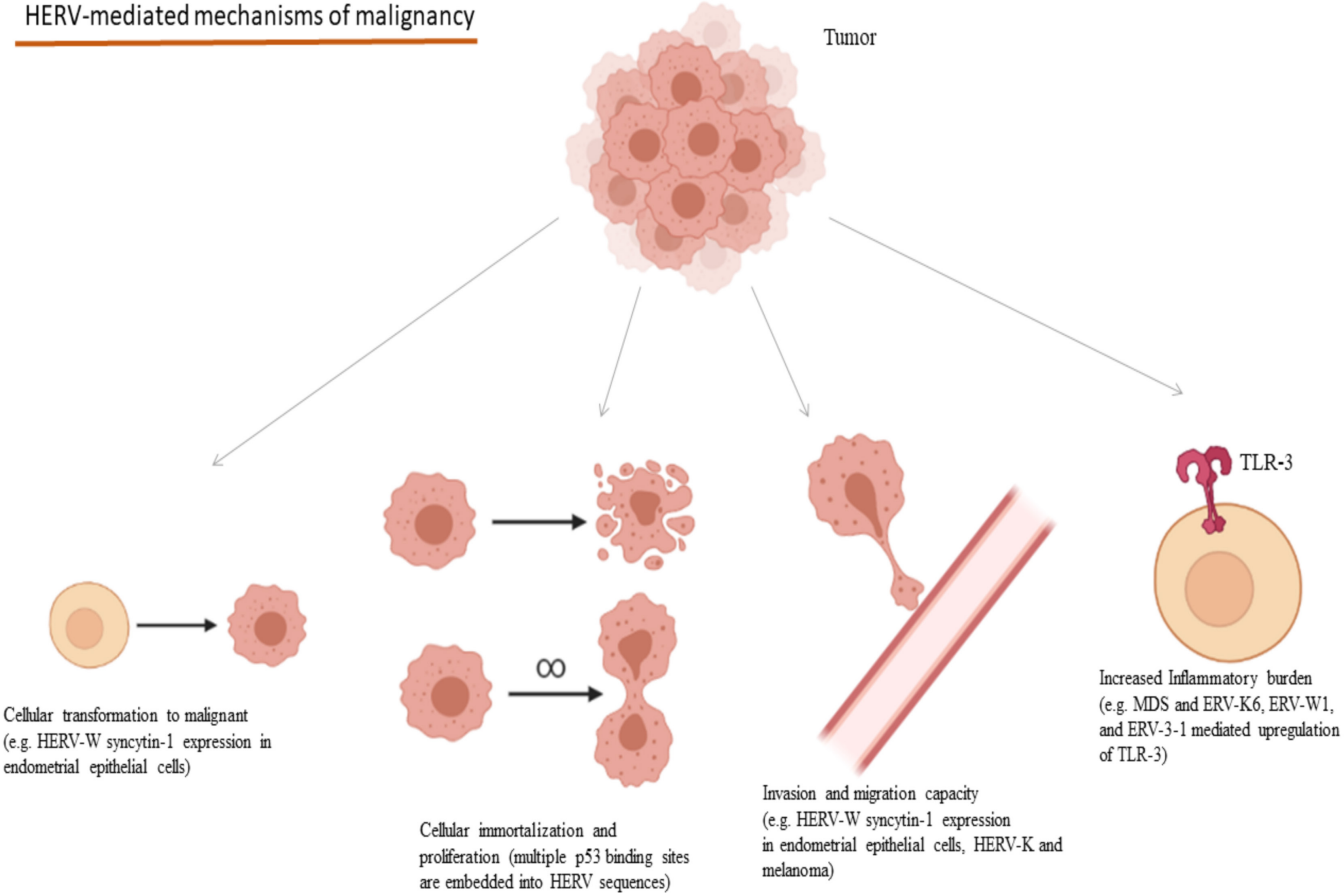
Oncogenic mechanisms mediated through HERVs and selected examples of their implications in cancers. (HERV, Human Endogenous Retrovirus; MDS, Myelodysplastic Syndrome; TLR, Toll-like Receptor). Created in BioRender.com.

HERV expression has also been considered as a potential prognostic factor in the disease course and survival in many cancers and their presence affects these characteristics in multiple manners. Colorectal cancer tissues characterized by increased endogenous retroviral expression combined with a CD8(+) infiltration, have been linked to increased inflammatory burden and unfavorable prognosis in terms of treatment response and disease recurrence (217). HERV-W *syncytin-1* expression in endometrial epithelial cancer induces cell transformation to mesenchymal, cell proliferation as well as cell migration and adhesion increasing the metastatic potential of the tumor (199). Likewise, HERV-K induction in malignant melanoma increases the melanoma cell stemness and transformation potential, invasion and migration capacity, increasing its overall malignant dynamic (200). Regarding melanoma, HK2 Rec protein is implicated in the regulation of Melanocyte Inducing Transcription Factor (MIFT) and MIFT, which in turn, causes the upregulation of HERV-K LTR and proviral expression, implying a loop between MIFT and HERV-K transcription in the development of the disease (218, 219). HK2 Np9 accessory protein has been implicated in the development and characteristics of teratocarcinoma. *In vitro* data suggest that Np9 may have oncogenic potential, as its depletion decreased the malignant potential of the teratocarcinoma NCCIT cell line and made these cells more susceptible to bleomycin and cisplatin (220). Mechanisms of the contribution of HERVs in the development of the hallmarks of cancer are summarized in **Figure 4**.

On the other hand, a HERV expression-based score for epithelial ovarian cancer was built, and higher scores were associated with longer survival and better prognosis (207). *In vitro*, it was shown that DNA methyltransferase inhibition led to ERV-mediated interferonogenic reactions, that activated CD8(+) cytotoxic cells that induced epithelial cancer cell killing (207). Interestingly, clear cell Renal Cell Carcinoma (ccRCC) demonstrates overexpression of a retroviral antigen derived from a member of the HERV-E family (CT-RCC HERV-E or ERVE-4), compared to healthy tissues, which can be identified by antigen-specific CD8(+) T -cells, as has been reported in a patient who experienced regression of metastatic ccRCC after allogeneic hematopoietic stem cell transplantation (221). More specifically, transcription of the entire env gene of both domains (surface and transmembrane) of CT-RCC HERV-E is detected in ccRCC cells and CD8(+) T-cells were capable to identify HERV-E expressing ccRCC tumor cells that are HLA-A*0201 positive (222). However, CT-RCC HERV-E expression does not appear to take place solely in ccRCC cells, as it has been recognized to have translational capacity, also, in lymphoblastic cell lines, which may indicate the potential this HERV-E family member to be expressed in malignant cells in general (223).

Among all cancers analyzed by Smith et al. with respect to their HERV expression patterns, ccRCC demonstrated the greatest number of HERVs with potential prognostic value (223). Interestingly, a recombinant integration of HERV-E (chromosome 19, positions 29097422-29106221) that has been named as hERV 4700, which demonstrated the highest level of expression in ccRCC tissues compared to healthy tissues, demonstrated strong evidence of translation in ccRCC tissues (223). More specifically, predicted hERV 4700 epitopes in ccRCC tumours originate from gag, pol and env sequences, and infiltrating T-cells specific for the gag- and pol-derived epitopes of hERV 4700 are described in ccRCC tissues, while these hERV 4700-specific T-cells are hardly present in PBMC populations of healthy donors (223). Thus, hERV 4700-epitopes are able to elicit T-cell responses against ccRCC cells, and hERV 4700 expression can be considered a biomarker of responsiveness of ccRCC tumors to checkpoint inhibitor anticancer treatments (223). Bearing in mind that ccRCC is a malignancy with a poor prognosis, further studies on the role of HERV-E family are required, as this HERV may provide the scientific community with promising novel cancer immunotherapy targets.

## Conclusion

Our understanding of the role of free-living and endogenized human viruses in the development of cancer has greatly improved over the last few years. The revolution of high throughput sequencing technologies has allowed us to reveal hidden interactions, and we now see deeper and more perplexing relationships within the network of interactions of humans and a better-understood variety of viral forms. It has, thus, become clearer that the human-virus evolutionary gameplay is mainly driven by the pleiotropic effects of the viral ecology on the human host. In the case of ERVs, their intricate evolutionary history and the multiplicity of their roles in the pathogenesis of human malignancy warrants further investigation which is promising for the provision of novel diagnostic and therapeutic perspectives. However, even though viruses remain an established cause of cancer, their molecular pathways remain to be disentangled. As we shed light on these pathways, we foresee that a better understanding of cancer will emerge as well as novel avenues for therapeutic intervention.

## Author Contributions

KK: Writing- Original draft preparation, Writing- Reviewing and Editing. MI: Conceptualization, Writing- Original draft preparation, Writing- Reviewing and Editing. VS: Writing- Reviewing and Editing. PL: Conceptualization, Writing- Reviewing and Editing. GM: Conceptualization, Writing- Reviewing and Editing, Supervision. All authors contributed to the article and approved the submitted version.

## Conflict of Interest

The authors declare that the research was conducted in the absence of any commercial or financial relationships that could be construed as a potential conflict of interest.

## Publisher’s Note

All claims expressed in this article are solely those of the authors and do not necessarily represent those of their affiliated organizations, or those of the publisher, the editors and the reviewers. Any product that may be evaluated in this article, or claim that may be made by its manufacturer, is not guaranteed or endorsed by the publisher.
